# Editorial: Highlights of the 1st Fun-Ex Conference: evolution, biodiversity, taxonomy and genomics of extremophilic and extremotolerant fungi

**DOI:** 10.3389/ffunb.2024.1538341

**Published:** 2024-12-23

**Authors:** Claudia Coleine, Ramón Alberto Batista-García

**Affiliations:** ^1^ Department of Ecological and Biological Sciences, University of Tuscia, Viterbo, Italy; ^2^ Centro de Investigación en Dinámica Celular, Instituto de Investigación en Ciencias Básicas y Aplicadas, Universidad Autónoma del Estado de Morelos, Cuernavaca, Morelos, Mexico; ^3^ Departamento de Biología Animal, Biología Vegetal y Ecología, Facultad de Ciencias Experimentales, Universidad de Jaén, Jaén, Spain

**Keywords:** extremophilic fungi, polyextremotolerant fungi, fungal diversity, extreme habitats, fungal adaptations, 1st Fun-Ex Conference

Extremophilic and extremotolerant fungi inhabit some of the most challenging environments on Earth, thriving where few organisms can survive. From deserts and glaciers to hypersaline lakes and acidic rivers, these fungi exhibit remarkable adaptations that allow them to withstand extremes of dryness, temperature, pH, salinity, radiation, and oxidative stress. The 1st Fun-Ex Conference was a pivotal moment for the scientific community, providing a platform to discuss these extraordinary organisms’ evolutionary, ecological, and biotechnological dimensions. Held to foster interdisciplinary collaboration, the conference successfully highlighted the diversity and adaptability of extremophilic fungi while sparking new ideas for future research.

This Research Topic sought to address three key objectives: (1) to advance our understanding of the biodiversity and adaptations of extremophilic and polyextremotolerant fungi; (2) to explore their roles in extreme ecosystems and their potential impacts, whether beneficial or detrimental; and (3) to uncover practical applications of these fungi in bioremediation, bioenergy, and materials science. The six papers published as part of this Research Topic have met these goals and revealed intriguing new insights into fungal resilience, metabolic versatility, and ecological significance.

## 1 Meeting the objectives: insights and innovations

### 1.1 Fungal adaptations to oxidative stress

Understanding the mechanisms by which fungi manage oxidative stress is critical to uncovering their survival strategies in extreme environments. Research on *Rhodotorula mucilaginosa*, a carotenoid-producing yeast, provided important insights into how oxidative metabolism and carotenoid production intersect. The study demonstrated that this yeast produces carotenoids to mitigate reactive oxygen species (ROS) in oxidative phosphorylation conditions. However, these carotenoids can paradoxically become prooxidants under specific conditions, as observed when interacting with free radicals. This dual role underscores the complexity of fungal adaptations and raises new questions about the trade-offs between protective and potentially harmful responses to oxidative stress (Mosqueda-Martínez et al.).

### 1.2 The multifaceted role of fungal melanin

Melanin is an essential biomolecule, which is widespread in the Tree of Life, that contributes to the stress tolerance of many fungi, particularly those living in extreme conditions. A detailed investigation into the melanin of *Exophiala mesophila* revealed its unique physicochemical and electrochemical properties. This study showed that melanin from this metallotolerant fungus could effectively bind heavy metals such as Cr^6+^, highlighting its potential for environmental remediation. The research also demonstrated the involvement of semiquinone/hydroquinone redox transformations, suggesting that fungal melanin might play a role in microbial electron transfer processes. These findings emphasize the biotechnological potential of fungal melanin, particularly in fields like bioremediation and sustainable materials science (Medina-Armijo et al.).

### 1.3 Fungal diversity in extreme habitats

Fungi biodiversity in extreme environments is often underappreciated, but several contributions to this topic showcase their ecological significance. A study of fungal communities associated with the petroglyphs of Israel’s Negev Desert revealed a specialized mycobiota adapted to the arid environment. Rock-specialized mycobiota consisting of extremotolerant microcolonial fungi and lichens were identified as key players in these habitats. However, their role in biodeterioration threatens cultural heritage, illustrating the dual nature of extremophilic fungi as both resilient organisms and potential agents of damage (Rabbachin et al.).

The Great Salt Lake in Utah offered another fascinating glimpse into fungal biodiversity. This hypersaline environment, characterized by salt-saturated waters and extreme gradients, hosts a rich fungal community that includes genera such as *Acremonium*, *Cladosporium*, and *Wallemia*. These fungi occupy diverse niches, contributing to nutrient cycling and providing ecosystem services like salinity stress alleviation for other organisms. The study’s integration of cultivation and environmental DNA techniques highlighted the importance of fungi in maintaining the ecological balance of extreme ecosystems (Parrott and Baxter).

### 1.4 Genomic perspectives on fungal extremophilicity

Comparative genomics is a powerful tool for understanding the molecular basis of fungal adaptations. The genome analysis of *Cryomyces antarcticus*, a highly melanized endolithic fungus from McMurdo Dry Valleys in Antarctica, provided valuable insights into the genetic strategies that underpin its survival in extreme cold and radiation. Expanded gene families associated with stress tolerance and increased GC content in coding regions were identified as crucial factors in its extremophilic nature. Intriguingly, the study also suggested the presence of alternative haplotypes, raising questions about the organism’s reproductive strategies and genome plasticity. These findings lay the groundwork for further research into the genomic signatures of extremophilic fungi and their potential applications (Gomez-Gutierrez et al.).

### 1.5 Seasonal diversity and heavy metal tolerance in acidic habitats

The Río Tinto basin in southwest Spain provided a unique setting to study the seasonal diversity of fungal communities in an acidic and metal-rich environment. The study identified representatives from Ascomycota, Basidiomycota, and Mucoromycota, with some isolates demonstrating remarkable tolerance to toxic heavy metals such as Cu²⁺ and Cd²⁺. These findings expand our understanding of fungal diversity in acidophilic habitats and highlight their potential for heavy metal bioremediation. The correlation of fungal diversity with seasonal changes in physicochemical parameters underscores these communities’ dynamic nature and adaptability to fluctuating conditions (Oggerin et al.).

## 2 Themes and emerging insights

The research presented at the Fun-Ex Conference underscores several recurring themes:

### 2.1 Complexity of fungal adaptations

Fungal responses to extreme environments involve unique biochemical and genetic mechanisms. The dual roles of carotenoids and melanin illustrate the delicate balance between protective adaptations and potential trade-offs.

### 2.2 Ecological importance

Extremophilic fungi play critical roles in nutrient cycling, ecosystem stability, and the facilitation of life in harsh conditions. However, their activities can also pose risks, such as the biodeterioration of cultural heritage or competition with native species in extreme habitats.

### 2.3 Biotechnological potential

The unique properties of extremophilic fungi, such as their ability to tolerate heavy metals or thrive in hypersaline conditions, hold promise for applications in environmental remediation, bioenergy, and sustainable material development.

## 3 Future directions and the role of the Fun-Ex Congress in advancing extreme mycology

The 1st Fun-Ex Conference has established a strong foundation for studying extremophilic fungi, setting the stage for both immediate advancements and long-term impacts in the field. Extremophilic fungi represent a frontier of biological research, offering insights into how life adapts and thrives under extreme conditions while presenting unparalleled opportunities for biotechnological innovation. Despite these advances, critical questions remain unanswered, and the field faces significant challenges that must be addressed through collective efforts and interdisciplinary collaboration.

One of the most pressing needs is to deepen our understanding of fungal adaptations at the molecular level. Future research should integrate multi-omics approaches, such as proteomics and metabolomics, with existing genomic studies to provide a more comprehensive view of how these fungi manage stress, maintain cellular stability, and metabolize substrates under extreme conditions. These integrated approaches will enable researchers to unravel complex biological processes, from the stabilization of DNA in high-salinity environments to the synthesis of protective compounds like carotenoids and melanin in response to radiation or oxidative stress.

The study of chaotolerant and chaophilic fungi, particularly in the context of their unique adaptations to chaotropic stress, represents a critical frontier in this effort. As this field marks its 10th anniversary (Zajc et al., 2014), it faces several key challenges, including limited understanding of the molecular mechanisms that enable these fungi to thrive in destabilizing conditions, such as high MgCl₂ concentrations. These fungi offer an exceptional model to explore the molecular and biochemical strategies that extend beyond typical extremotolerance, revealing how organisms stabilize cellular structures and enzymatic functions under conditions that disrupt water activity and protein folding. However, progress in this area is hindered by a lack of well-characterized species, inconsistent methodologies, and limited ecological data.

Expanding the scope of investigation to underexplored habitats also presents an exciting opportunity. While much has been learned from fungi in deserts, glaciers, and hypersaline lakes, extreme environments such as deep-sea hydrothermal vents, ultramafic soils, and even extraterrestrial analogs like Mars-like terrains remain largely uncharted. These habitats could harbor new fungal species, reveal novel adaptation strategies that challenge our understanding of biology, and open doors to innovative applications in extreme environments and habitability on Earth and beyond. Indeed, beyond terrestrial confines, research into extraterrestrial analogs, such as Mars-like terrains or the icy crusts of Europa, could push the boundaries of astrobiology. Fungi adapted to these conditions might provide models for life in extraterrestrial environments, offering clues to the resilience of life and informing planetary exploration missions. Collaborations with space agencies and planetary scientists could lead to innovative bioengineering projects, such as developing fungal-based biotechnologies for supporting life in space habitats.

Translating discoveries into practical applications is another critical avenue for future work. Extremophilic fungi hold immense promise for bioremediation, including detoxifying heavy metals, mitigating salinity stress in agriculture, and degrading pollutants in hostile environments. For instance, fungal melanin’s ability to bind heavy metals like Cr^6+^ highlights its potential for creating sustainable materials that address environmental challenges. Similarly, the resilience of fungal communities in ecosystems like the Great Salt Lake suggests their potential as bioinoculants to support plant growth in saline soils. However, scaling these applications requires bridging the gap between fundamental research and industry, with collaborations involving microbiologists, environmental scientists, engineers, and policymakers.

In this context, the Fun-Ex Congress is poised to play a pivotal role in advancing the study and application of extremophilic fungi. As the field grows, the congress should evolve into an international forum that fosters collaboration, promotes interdisciplinary research, and consolidates fragmented efforts in extreme mycology. By bringing together experts from diverse backgrounds, the Fun-Ex Conference can facilitate the integration of cutting-edge technologies, such as synthetic biology and artificial intelligence, into fungal research. These technologies could enhance our ability to predict fungal behavior, optimize their performance in industrial applications, and even engineer new strains with tailored functionalities.

Moreover, the Fun-Ex Conference provides a unique platform to address some of the field’s main challenges, such as the lack of standardized methodologies for studying extremophilic fungi and the need for more robust funding mechanisms to support long-term research. By serving as a hub for knowledge exchange and capacity building, the congress can ensure that the study of extremophilic fungi remains at the forefront of scientific innovation while highlighting their relevance to global challenges like climate change, food security, and sustainable development.

The 1st Fun-Ex Conference has shown that the study of extremophilic fungi is more than just an academic pursuit; it is a gateway to understanding life’s boundaries and harnessing its resilience for the benefit of humanity. By exploring the untapped potential of these resilient organisms, humanity can expand its understanding of life’s limits and unlock transformative solutions for a changing world. As this congress grows into a recurring event, it has the potential to transform extreme mycology into a cornerstone of biological research and innovation, guiding the next generation of scientists toward answering fundamental questions and applying their discoveries to address real-world challenges. By uniting the community and shaping a shared vision for the future, the Fun-Ex Conference can help unlock the full potential of extremophilic fungi, ensuring their impact on science and society for years to come.

## 4 Conclusion

The 1st Fun-Ex Conference marked a significant milestone in advancing our understanding of extremophilic and extremotolerant fungi. The diverse studies presented have deepened our knowledge of fungal adaptations, biodiversity, and ecological roles while opening new avenues for biotechnological applications. By fostering interdisciplinary collaboration and addressing critical gaps in knowledge, the Fun-Ex initiative is poised to shape the future of extreme mycology, ensuring that these remarkable organisms continue to inspire and inform both science and society.
